# A dual-targeting ruthenium nanodrug that inhibits primary tumor growth and lung metastasis via the PARP/ATM pathway

**DOI:** 10.1186/s12951-021-00799-3

**Published:** 2021-04-23

**Authors:** Yu Lu, Di Zhu, Lin Gui, Yuanming Li, Wenjing Wang, Jiawang Liu, Yuji Wang

**Affiliations:** 1 Department of Medicinal Chemistry, College of Pharmaceutical Sciences of Capital Medical University, 10 Xi Tou Tiao, You An Men, Beijing, 100069 People’s Republic of China; 2 Beijing Area Major Laboratory of Peptide and Small Molecular Drugs, Engineering Research Center of Endogenous Prophylactic of Ministry of Education of China, Beijing Laboratory of Biomedical Materials, Beijing, 100069 People’s Republic of China; 3 Minimally Invasive Tumor Therapies Center, Beijing Hospital, National Center of Gerontology, Institute of Geriatric Medicine, Chinese Academy of Medical Sciences, Beijing, 100730 People’s Republic of China; 4 Beijing Institute of Hepatology, Beijing Youan Hospital, Capital Medical University, Beijing, 100069 People’s Republic of China; 5 Medicinal Chemistry Core, The University of Tennessee Health Science Center, 579 College of Pharmacy Building, 881 Madison Avenue, Memphis, TN 38163 USA

**Keywords:** Ruthenium, Self-assembly, Cell cycle, Apoptosis, Antitumor, Antimetastatic

## Abstract

**Background:**

Many studies have found that ruthenium complexes possess unique biochemical characteristics and inhibit tumor growth or metastasis.

**Results:**

Here, we report the novel dual-targeting ruthenium candidate **2b**, which has both antitumor and antimetastatic properties and targets tumor sites through the enhanced permeability and retention (EPR) effect and transferrin/transferrin receptor (TF/TFR) interaction. The candidate **2b** is composed of ruthenium-complexed carboline acid and four chloride ions. In vitro, **2b** triggered DNA cleavage and thus blocked cell cycle progression and induced apoptosis via the PARP/ATM pathway. In vivo*,* **2b** inhibited not only Lewis lung cancer (LLC) tumor growth but also lung metastasis. We detected apoptosis and decreased CD31 expression in tumor tissues, and ruthenium accumulated in the primary tumor tissue of C57BL/6 mice implanted with LLC cells.

**Conclusions:**

Thus, we conclude that **2b** targets tumors, inhibits tumor growth and prevents lung metastasis.
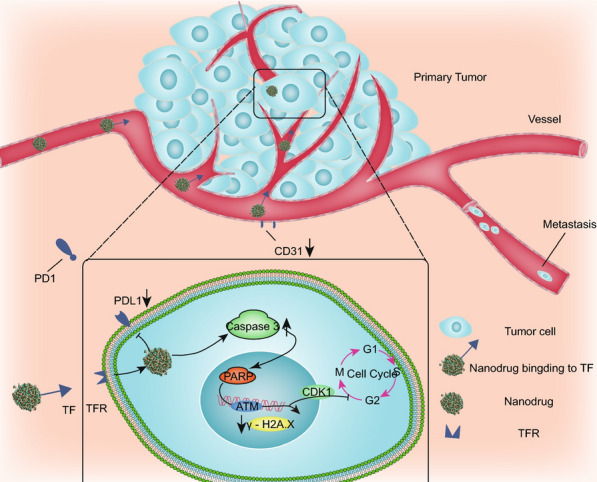

## Background

Metal-based antitumor drugs, such as cisplatin, have been extensively studied over the past few decades [1]. Research shows that ruthenium complexes inhibit tumor growth or metastasis due to their unique biochemical characteristics [2–10]. In a phase I clinical study, NAMI-A inhibited lung metastasis [11–13] and generation of peripheral blood vessels in tumor tissues [14, 15] but did not suppress primary tumor growth.

Carboline alkaloid is an active ingredient extracted from traditional Chinese medicine. Our group has conducted in-depth research on its antitumor and other activities [16, 17]. Many studies have combined carboline and its derivatives with ruthenium to obtain a series of antitumor active compounds [18–22]. However, these complexes have not been reported to inhibit lung metastasis.

Although platinum (IV) prodrugs have great potential to kill tumor cells and reduce side effects [23, 24], few studies have focused on ruthenium (IV) complexes. Vilaplana et al. [25] designed and synthesized the first ruthenium (IV) complex with antitumor effects. The cytotoxic complex was likely transported into tumor sites via transferrin (TF) because halides are easily replaced at the TF binding site [26]. TF is a glycoprotein that controls the extracellular iron level. TF reversibly binds polyvalent ions, including iron, copper, cobalt, and ruthenium [27]. Transferrin receptors (TFRs) are expressed in both normal and cancer tissues. However, TFR expression in cancer cells can be 100-fold higher than that in normal cells [28, 29].

**Fig. 1:**
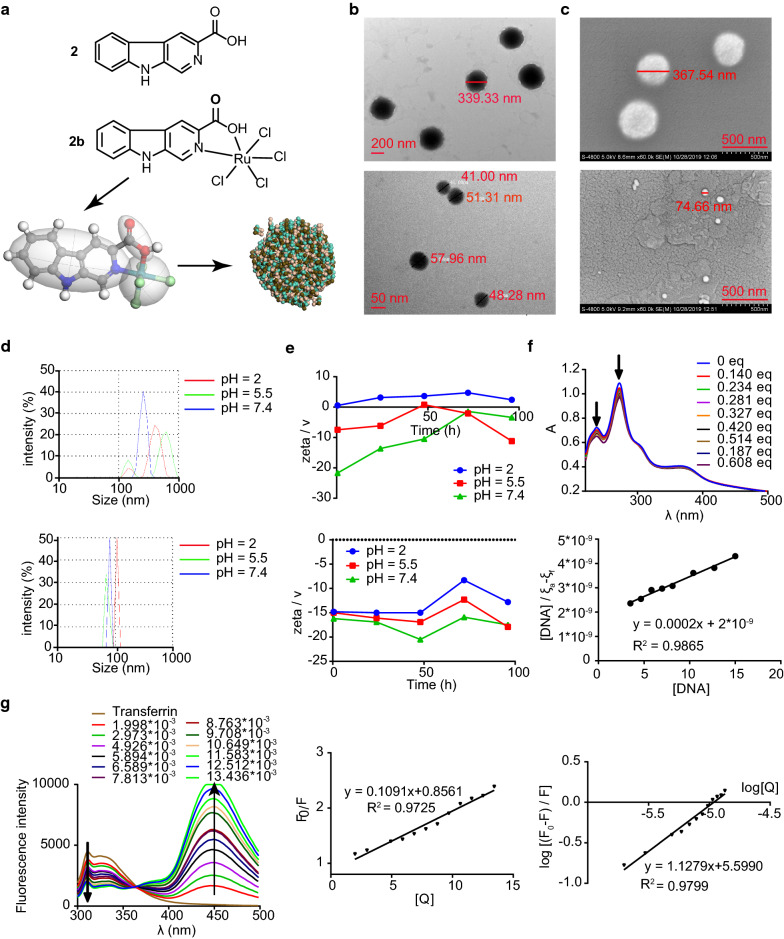
Physicochemical characterization of **2b**. **a** Structure of **2** and **2b** and mesoscale simulation of 2b. **b** TEM image of the Ru ligand **2** (up) and complex **2b** (down). **c** SEM image of the Ru ligand **2** (up) and complex **2b** (down). **d** Size of **2** (up) and **2b** (down) at different pH values. **e** Zeta potentials of **2** (up) and **2b** (down) at different pH values within 96 h (detected at 0.5 h, 24 h, 48 h, 72 h and 96 h). **f** UV spectra of **2b** with different concentrations of ctDNA. **g** Fluorescence emission spectra of hTF (0.4 μM, λ_ex_ = 280 nm) with different concentrations of **2b**. Classical Stern–Volmer equation and modified Stern–Volmer equation plots showing tryptophan quenching in hTF

Our aim was to obtain a dual-targeted ruthenium complex with both antitumor and antimetastatic properties. This complex targets tumor sites through both the enhanced permeability and retention (EPR) effect and TF/TFR interaction. We designed and synthesized the ruthenium complex 2b (ruthenium (IV)) (Fig. 1a). To test the hypothesis that chloridion plays an important role in tumor-targeting therapy, complex 2a (ruthenium (II)) was synthesized to be used for comparison. In vitro, we compared the cytotoxic effects of ligand **2** and complexes **2a** and **2b** on various cancer cell types and normal cells using MTT assays. We determined the self-assembly and interaction of **2b** with DNA and TF to explore dual-targeting functions. We also determined the localization of ruthenium via inductively coupled plasma mass spectrometry (ICP-MS), protein expression in A549 cancer cells via western blotting, and effects of **2b** on apoptosis and the cell cycle to further explain its mechanism. In vivo, we evaluated the effects of **2b** on primary tumor growth and lung metastasis in C57BL/6 mice implanted with Lewis lung carcinoma (LLC) cells. We also assessed apoptosis in tumor tissues via terminal deoxynucleotidyl transferase dUTP nick end labeling (TUNEL) and platelet endothelial cell adhesion molecule-1 (CD31) expression via immunohistochemistry.

## Results

### Characterization and nanoscale self-assembly properties of Ru(IV) complexes

We deduced the compound structures (Fig. 1a) from the characterization information obtained from mass spectroscopy (MS), Fourier-transform infrared spectroscopy (FTIR), and ^1^H NMR and ^13^C NMR spectroscopy (Additional file 1). The NMR spectra and FTIR data indicated coordination of ligand 2 to the metal precursor. A slight downfield shift was observed compared to ligand 2, which was consistent with results reported in the literature [30]. In addition, the peaks in the FTIR spectrum moved to the low wavenumber region. Specifically, the peak at 1720 cm^−1^ (υ_C=O_) in the FTIR spectrum of ligand **2** shifted to 1621 cm^−1^ and changed to a broad peak in the FTIR spectrum of complex **2b**. The final complexes were characterized using mass spectra.

Complexes **2a** and **2b** were air stable and water soluble, while the carboline derivative 2 was only slightly water soluble (Photographs of the compound aqueous solution are shown in Additional file 1: Figure S14). This indicates that the addition of ruthenium increased the water solubility of the complexes.

We first simulated the self- assembly of complex **2b** using a Materials Studio molecular dynamics simulation technique and found that **2b** formed a spherical structure (Fig. 1a). Next, we compared the nanoscale self-assembly properties of ligand **2** and complex **2b** from three aspects: shape and size were observed via transmission electron microscopy (TEM) and scanning electron microscopy (SEM) (Fig. 1b, c), the average hydrodynamic diameters were measured with a dynamic light-scattering (DLS) analyzer (Fig. 1d), and the zeta potential was determined over 96 h (Fig. 1e). TEM and SEM images showed that both **2** and **2b** have a spherical structure; the diameter of **2b** is approximately 50 nm; that of ligand **2** is larger, at approximately 300 nm. The average hydrodynamic diameters of **2b** and **2** are approximately 100 nm and 400–600 nm, respectively. We think that the smaller particle size of the **2b** nanoparticles is associated with an increase in solubility and that the addition of hydrophilic charged groups further increases solubility in water [31, 32]. The particle size in solution was found to be slightly larger than that in the solid state and changed slightly at different pH values.

The zeta potential of **2b** essentially remained stable for 96 h, while the zeta potential of **2** gradually fell to 0 within 96 h. This indicates that the **2b** nanoparticles were stable and dispersed in aqueous solution over 96 h. The zeta potential of **2** and **2b** are approximately − 10 mV and − 15 mV, respectively, at pH 7.0. The negative zeta potential is likely due to the negative charge of the COO^−^ groups [33]. As the pH decreased, the zeta potential of **2b** changed slightly but that of **2** decreased substantially. At a low pH of 2.0, **2** even showed a positive zeta potential but showed negative zeta potentials at higher pH values. This is consistent with previous nanoparticle studies [34, 35].

### Human transferrin (hTF) binding

We studied the binding ability of human transferrin (hTF) to complex **2b** by measuring fluorescence quenching of hTF [7, 27, 36]. With the addition of the Ru complexes, the tryptophan fluorescence peak (315 nm) decreased, whereas the fluorescence peak (450 nm) of complex **2b** increased (Fig. 1g). We calculated several constants representing the binding ability measured at 315 nm: Stern–Volmer quenching constant (K_sv_, L mol^−1^), biomolecular quenching rate constant (*K*_*q*_, L mol^−1^ s^−1^), binding constant (*K*_*b*_, L  mol^−1^) and the number of binding sites (*n*) (Table 1). The K_q_ value (Table 1) suggested that the fluorescence quenching process was static. The number of binding sites *n* and binding constant *K*_*b*_ (Table 1) indicated that complex **2b** could bind stably to hTF.

**Table 1:** The constant of K_sv_, *K*_*q*_, *K*_*b*_ and the number of binding sites (*n*) for **2b** and hTF

	K_sv_ * 10^5^ (L mol^−1^)	*K*_*q*_ * 10^13^ (L mol^−1^ s^−1^)	Number of binding sites (*n*)	*K*_*b*_ * 10^5^ (L mol^−1^)
**2b**	1.09	1.89	1.1	3.97

The FTIR spectrum of hTF in the presence of complex **2b** clearly confirmed binding of hTF to **2b** (Additional file 1: Figure S14). Upon addition of complex **2b**, the peak of hTF at 1560 cm^−1^ shifted to higher wavenumbers and reached 1593 cm^−1^, with an increase in the **2b** content and thus a decrease in the hTF/**2b** ratio. The hTF peak at 3442 cm^−1^ also shifted to higher wavenumbers and reached 3499 cm^−1^.

### DNA binding

**Fig. 2:**
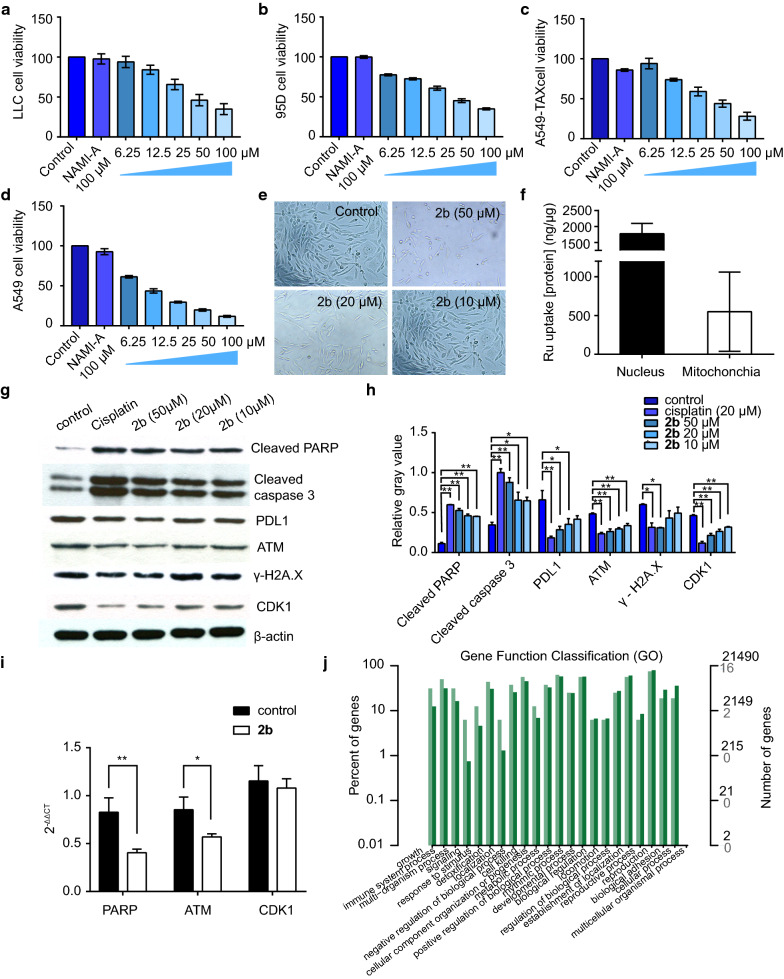
In vitro activity of **2b**. **a**–**d** Viability of LLC, 95D, A549-TAX and A549 cancer cells treated with different concentrations of **2b** and 100 μM NAMI-A. The concentrations of **2b** were 6.25, 12.5, 25, 50, and 100 μM. **e** Morphological changes in cells in the control and administration groups incubated with 5% CO_2_ at 37 °C for 48 h. **f** Intracellular uptake of Ru in the 20 μM group. **g**, **h** Western blotting image and relative gray value analysis of proteins in A549 cells treated with **2b**. **i** Quantitative real-time PCR results in A549 cells treated with **2b**. **j** Gene function classification (GO) of **2b**

Using ultraviolet–visible (UV) spectroscopy quenching of **2b,** we studied the ability of DNA to bind to complex **2b** [37]. With the addition of circulating tumor DNA (ctDNA) in PBS (pH 7.4), the UV absorption curve of 2b (12.5 μM) in solution decreased (Fig. 2f). We calculated the binding constant of **2b** with ctDNA to be 1.0 × 10^5^. The interaction between DNA and complex **2b** was also confirmed by FTIR spectra. Both peak I and II shifted to lower wavenumbers upon the 2b addition.

### In vitro cytotoxicity study

We evaluated the effects of **2**, **2a**, **2b** and **NAMI-A** on four lung cancer cell lines (LLC, 95D, A549-TAX and A549), a mouse sarcoma cell line (S180) and a normal liver cell line (L02; IC_50_ values in Additional file 1: Table S1). As reported in the literature [14], NAMI-A exhibited no cytotoxic effects in vitro*,* and in all cell lines, the IC_50_ was more than 100 μM. **2b** had similar effects on LLC, 95D and A549-TAX cancer cells, and the IC_50_ values were all approximately 50–60 μM (Fig. 2a–c). The killing effect of **2b** on A549 cells was better than that on the other cancer cell lines; the IC_50_ of **2b** in A549 cells was 10–20 μM (Fig. 2d), which was 4–5 times more effective than that in the other three cancer cell lines. In addition, **2b** killed more cancer cells within the same time than ligand **2** (Additional file 1: Table S1). Consequently, we chose A549 cells to observe the shape of cells in the control and administration groups (Fig. 2e). The figures show that A549 cells were fusiform under normal conditions, while the cells treated with **2b** decreased in size and became round.

### Cellular uptake

We chose A549 cells to study the distribution of **2b** in cells [38–41]. According to the manufacturer’s instructions, we separated the nuclei and mitochondria via centrifugation and detected the ruthenium content using ICP-MS (Fig. 2f). The results showed that ruthenium was mainly concentrated in the nucleus, with a nuclear content more than twice as high as the mitochondrial content.

### Effect on protein expression in A549 cells

We determined the expression levels of a series of proteins, including cleaved poly (ADP-ribose) polymerase (PARP), cleaved caspase 3, ataxia-telangiectasia mutated (ATM), gamma H2A histone family member X (γ-H2A.X), CDK1 and programmed cell death 1 ligand 1 (PDL1), in A549 lung cancer cells via western blotting [20, 21] (Fig. 2g, h). Compared with that in the normal group, cleaved PARP and cleaved caspase 3 expression increased significantly after incubation with **2b**; ATM, γ-H2A.X and CDK1 expression decreased in a concentration-dependent manner. No significant differences were observed between the high dose of **2b** and cisplatin in the expression of these proteins. On the other hand, **2b** also decreased expression of the immunosuppressive-related protein PDL1, suggesting that **2b** enhances the response of tumor cells to immune cells.

### The cell cycle and apoptosis

We studied the effect of **2b** on the cell cycle using flow cytometry [7, 22]. Cells were divided into G_0_/G_1_, S, and G_2_/M phases according to the fluorescence intensity of propidium iodide (PI). With an increasing concentration of **2b**, the number of cells in G_0_/G_1_ phase decreased gradually, whereas the number of cells in G_2_/M phase increased gradually; the number of cells in S phase did not change significantly. Hence, we concluded that **2b** is able to stall cells in G_2_/M phase.

**Fig. 3:**
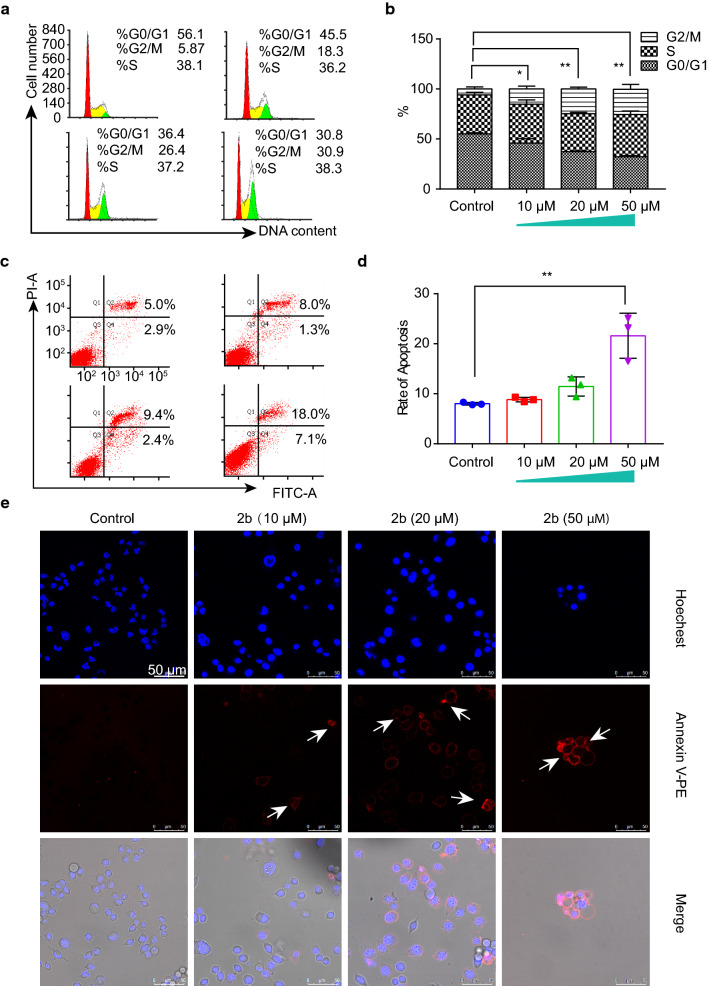
Effect of the Ru complex **2b** on cell cycle and apoptosis. **a**, **b** Effect of **2b** on the cell cycle. **c**, **d** Effect of **2b** on cell apoptosis (Annexin V-FITC/PI, assessed via flow cytometry). **e**, **f** Effect of **2b** on cell apoptosis (Annexin V-PE/Hoechst, observed with confocal microscopy)

We grouped the cells and determined the proportion of apoptotic cells (Q2 and Q4 areas) according to the fluorescence intensity of Annexin V-fluorescein isothiocyanate (FITC; Fig. 3c, d). With an increase in the concentration of **2b**, the number of cells in Q2 and Q4 gradually increased. We also observed apoptotic cells using laser confocal microscopy (Fig. 3e). The cell membranes of apoptotic cells were dyed red with Annexin V-phycoerythrin (PE), and the color of the nuclei was darker and brighter than that of normal cells. Thus, **2b** appeared to cause apoptosis.

### *Effect on tumor growth and metastasis *in vivo

**Fig. 4:**
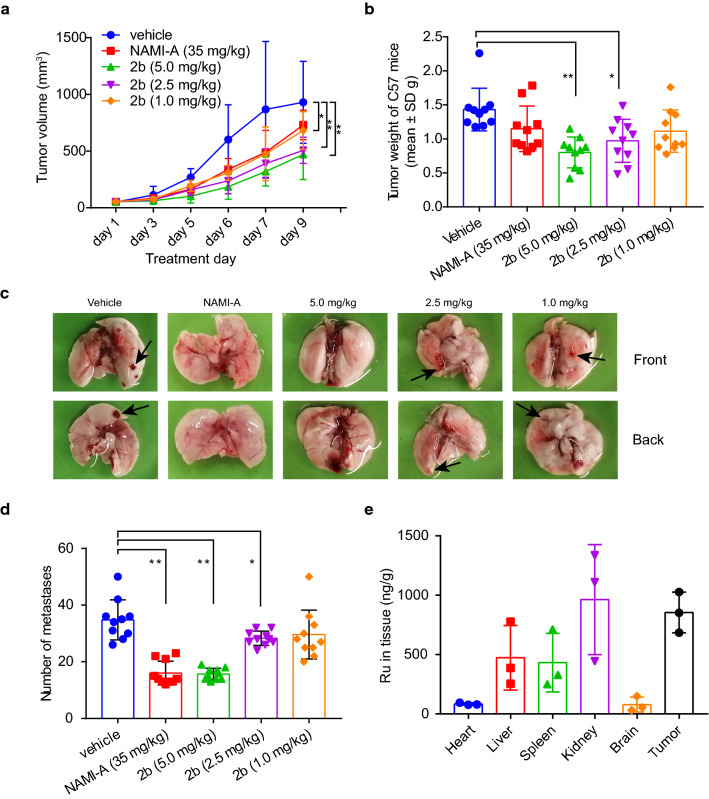
Effect of the Ru complex **2b** on LLC tumor growth and metastasis in C57BL/6 mice. **a** Dose-dependent volume curve of LLC tumors. **b** Tumor weights in treated C57BL/6 mice. **c** Representative images of metastasis in the lung. **d** The number of metastases. **e** Body distribution of **2b** (5.0 mg/kg), represented by the mean ± SD (ng) of ruthenium per g of organ, n = 6

We studied the effect of **2b** on primary tumor growth and lung metastasis in C57BL/6 mice implanted with LLC cells [13, 42]. Figure 4a, b show that the volume and weight of the primary tumors decreased in the mice receiving 5.0 mg/kg and 2.5 mg/kg **2b** and with increasing administration concentrations of **2b**. Figure 4c, d shows that the number of lung metastases decreased in the mice receiving 5.0 mg/kg and 2.5 mg/kg **2b** compared with the control group. No significant differences were observed between NAMI-A (35.0 mg/kg) and **2b** (5.0 mg/kg) in inhibiting primary tumor growth and lung metastasis.

### Organ weight and body distribution

Platinum agents can decrease organ weights [1]. The weights of the liver, kidney and spleen from mice treated with cisplatin were significantly lower than those of organs from mice in the vehicle group (Additional file 1: Figure S11). However, organ weights in the **2b** treatment groups and the vehicle group were not significantly different. We also determined the distribution of **2b** in the organs of C57BL/6 mice [43]. Figure 4e shows that the ruthenium content in the kidney and tumor was approximately two to threefold higher than that in the liver and spleen. This suggests that ruthenium was mainly excreted from the kidney and that **2b** can target tumors in vivo*.*

### Immunofluorescence and immunohistochemistry

**Fig. 5:**
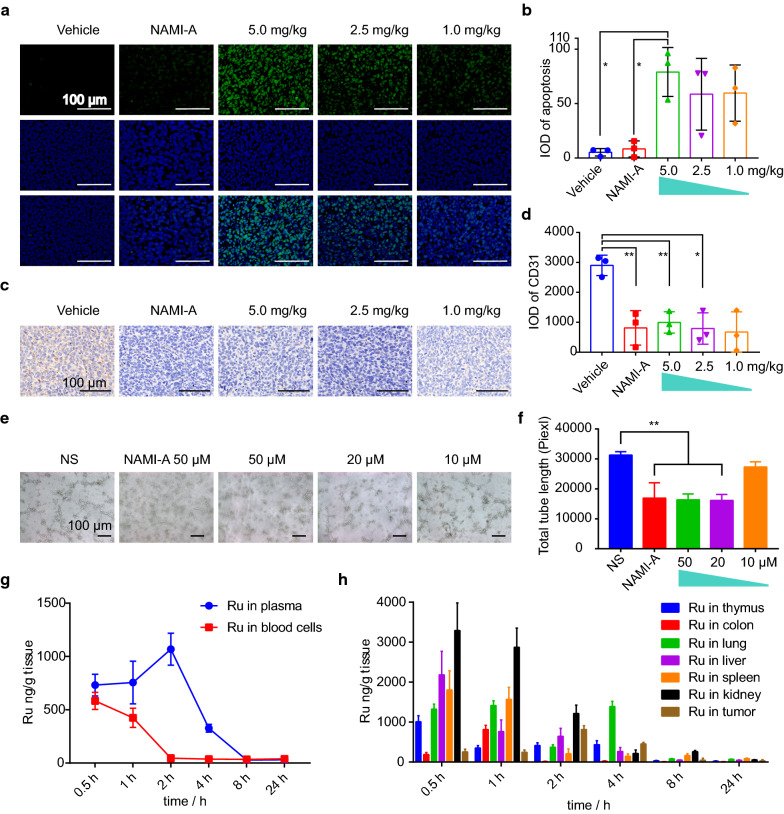
In vivo mechanism of **2b**. **a**, **b** Images and statistical analysis of apoptosis in tumor tissue determined by TUNEL. **c**, **d** Image and statistical analysis of CD31 expression in tumor tissue. **e**, **f** Image and statistical analysis of capillary tube formation. **h**, **i** Degradation of ruthenium over time in plasma, blood cells and organs after administration of a single **2b** dose of 5 mg/kg

Using TUNEL staining, we detected apoptosis induced by **2b** in tumor tissues [44]. As shown in Fig. 5a, b, the number of apoptotic cells (TUNEL-positive cells) in the NAMI-A group and vehicle group was not significantly different. However, the number of apoptotic tumor cells in the 5.0 mg/kg **2b** treatment group was significantly increased compared with that in the vehicle and NAMI-A groups. This suggests that drug-induced apoptosis may be one of the reasons that **2b** inhibits the growth of LLC tumors.

As CD31 is a marker of vascular endothelial cells, we evaluated the neovascularization of tumors according to CD31 expression [11–15]. As depicted in Fig. 5c, d, 35 mg/kg NAMI-A reduced CD31 expression and thus inhibited tumor metastasis. In addition, 5.0 and 2.5 mg/kg **2b** significantly reduced CD31 expression. We conclude that **2b** may exert its effects through the same mechanism as NAMI-A because they both inhibit tumor neovascularization to suppress tumor metastasis.

### Anti-angiogenesis in vitro

Capillary tube formation assays were used to assess the angiogenic activity of **2b** [14, 45–47]. HUVECs were seeded into a Matrigel-coated 24-well plate and treated with 50 μM **NAMI-A** and 50 μM, 20 μM, or 10 μM **2b** for 6 h. As shown in Fig. 5e, f, **NAMI-A** and **2b** decreased tube formation at 6 h. In addition, **2b** inhibited capillary tube formation in a dose-dependent manner.

### Degradation over time and toxicity studies of nanoparticles in vivo

Several clinical and preclinical data have suggested that Ru nanoparticles can be degraded over time in the body and excreted from the body [48]. The Ru content in the blood and organs was measured via ICP-MS at 6 different time points after administration of 5 mg/kg **2b**. At 30 min after administration, the Ru was distributed evenly in plasma and blood cells and reached a maximum plasma concentration at 2 h after administration, while the content in blood cells decreased to almost zero (Fig. 5h). As shown in Fig. 5i, the Ru content decreased gradually over time, indicating that the liver and kidneys continuously metabolized the nanoparticles. At 24 h after administration, almost all the Ru was eliminated from the body. The maximum Ru concentration in tumors was reached after 2 h. At 4 h, the Ru was mainly detected in the tumor and lung, and most of the Ru in normal organs had been excreted.

In the toxicity study, the heart, liver, spleen and kidney weights were significantly reduced in the cisplatin group, while the weights of organs in the **2b** group did not change (Additional file 1: Figure S20A–E). This suggests that the elimination of Ru effectively reduced toxicity. To further analyze the toxicity of **2b**, we measured blood biochemical parameters and found no changes in serum alanine aminotransferase (ALT), aspartate aminotransferase (AST), blood urea nitrogen (Urea) or creatinine (Crea) levels.

## Discussion

The nanoscale self-assembly properties of small molecule drugs are important in tumor-targeting therapy [49, 50], and drug delivery systems based on TF/TFR are important [51]. The dual-targeting nanoscale self-assembled ruthenium complex **2b** was designed to compensate for the shortcomings of low solubility and poor targeting of carbolines. In vitro, we compared the cytotoxic effects of ligand **2** and complex **2a** and **2b** on different cancer cell types and normal cells using MTT assays. Only **2b** was able to selectively kill cancer cells. This partially validates the hypothesis that chloridion plays an important role in tumor-targeting therapy. The introduction of chloridion into carbolines yields amphiphilic structures to increase selectivity for cells. We determined the self-assembly properties and protein binding characteristics of **2b** to evaluate its targeting characteristics. **2b** remained stable for 96 h [52]; and the size of **2b** is 50–100 nm, which is more favorable for targeting tumors than ligand **2** based on the EPR effect [50]. In addition, **2b** strongly bound to TF [27]. Because tumor tissues have more TFRs than normal tissues, **2b** can target tumor tissues with TF as the vector [29, 53].

**Fig. 6:**
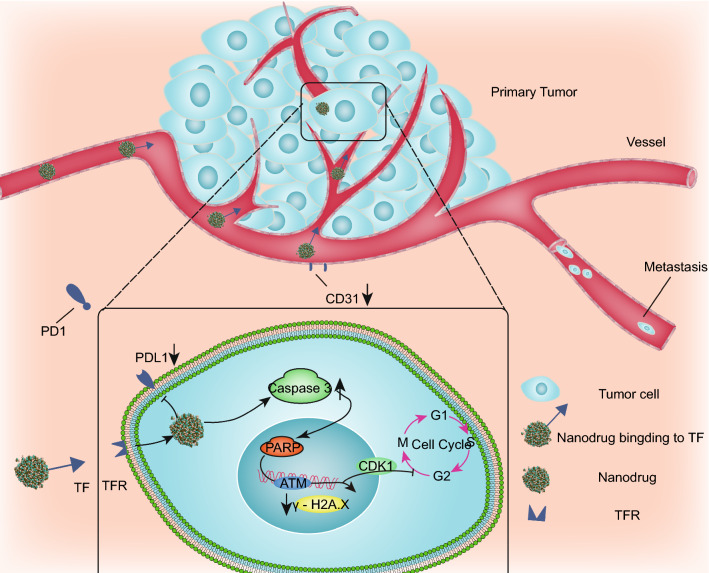
Mechanisms of complex **2b**

We studied the antitumor mechanism of **2b** in A549 cells (Fig. 6). MTT assays showed that **2b** was able to kill lung cancer cells. The cell uptake assay showed that ruthenium was mainly concentrated in the nucleus of A549 cells, which indicates that the primary site of action of **2b** is the nucleus and that **2b** can bind to DNA in vitro. Hence, we evaluated cell cycle and apoptosis and found that **2b** blocked cell cycle progression in G2/M phase and caused apoptosis. To further illustrate this mechanism, we detected expression of proteins in A549 cells and found substantial changes in cleaved caspase 3, cleaved PARP, ATM and CDK1 expression, whereas PDL1 expression decreased.

For in vivo study*,* we selected C57BL/C mice bearing LLC tumors to evaluate the antitumor and antipulmonary metastasis activity of the nanodrugs. Previously, we determined the effect of **2b** on PDL1 expression in A549 cells. PDL1 is an immunosuppressive protein located on the cell membrane. Some small molecule drugs can inhibit PDL1 expression, thus exposing tumor cells and reducing immune escape of tumor cells [54]. Considering the role of PDL1, we selected C57BL/C mice bearing LLC tumors to evaluate its biological activity. The results showed that 5 mg/kg and 2.5 mg/kg **2b** inhibited primary tumor growth and lung metastasis. Although **2b** inhibited PDL1 expression in vitro, no changes in PDL1 expression in tumor tissues were detected (Additional file 1: Figure S12), which may be the result of changes in the genome of tumor cells and may be influenced by biological factors. This needs to be studied further.

Assessment of the in vivo distribution of ruthenium showed that the Ru content in tumors accounted for 33.4% of the total content, indicating that **2b** had good tumor targeting ability. The results of TUNEL staining of tumor tissue revealed obvious apoptosis in tumor tissue in the 5 mg/kg **2b** treatment group, which was consistent with the in vitro experimental results. This suggests that **2b** may inhibit tumor growth by inducing apoptosis in tumor cells in vivo. Previous studies have shown that NAMI-A can reduce CD31 expression by inhibiting endothelial cell functions, thereby inhibiting tumor metastasis [11]. Immunohistochemical analysis of CD31 showed that CD31 expression was significantly lower in the 5.0 mg/kg and 2.5 mg/kg **2b** treatment groups than in the control group. In addition, capillary tube formation assays showed that **2b** inhibited endothelial cells from forming a capillary-like network on Matrigel in vitro. This indicates that **2b** might inhibit lung metastasis of tumor cells by inhibiting angiogenesis.

## Conclusions

The novel small molecule nanodrug candidate **2b** with dual targeting acts through the EPR effect and TF/TFR interaction in A549 cancer cells and LLC tumors in C57/BL6 mice*.* The advantages of the nanodrug are threefold, as follows. (1) The EPR effect and TF/TFR interaction with nanoparticles improve tumor targetability in vivo. The Ru content in tumors accounted for 33.4% of the total content (Fig. 4e). (2) Using ruthenium nanoparticles as a dual targeting drug delivery system significantly decreases the effective dose. The effective dose of **2b** in vivo was one-seventh that of **NAMI-A** and **KP1019** (Fig. 4b). In addition, (3) the nanodrug reduces the toxicity of drugs. As shown in Additional file 1: Figure S20, the body weight of ICR mice implanted with S180 tumors did not change in the 2b groups, but that in the cisplatin groups decreased. Furthermore, no significant changes in serum ALT, AST, Crea and Urea levels in comparison to the saline group on day 7 after **2b** administration were found, indicating that **2b** was not toxic to the mouse liver or kidneys. This study provides us with a new idea for combining passive and active targeting that is specific to ruthenium complexes.

## Methods

### Materials and reagents

Please refer to Additional file 1 for the materials and reagents used. All methods are recorded in Additional file 1 except the indicated experiments.

### Statistical analyses

Data are presented as the mean ± SD. Statistical significance was evaluated by one-way ANOVA using Prism 7.0 software. Significance was set at *P < 0.05; extreme significance was set at **P < 0.01.

### Nanoscale self-assembly properties

To predict its nanostructure, we defined **2b** as an amphiphilic molecule in molecular dynamics simulations using Materials Studio: Carboline—nonpolar group; carboxyl group and chloride—polar (and charged) groups. As described in reference [55], in a cubic box of 20 * 20 * 20 Å, the model of **2b** was randomly distributed with a density of 0.5 g cm^−3^. A 15 000 ps simulation was performed on this system at 298 K using the NVT ensemble.

To explore the nanoscale self-assembly properties of **2b** and its ligand **2** in solution and in solid state, a DLS particle size analyzer, TEM and SEM were applied. The nanoparticles were prepared via the self-assembly method [43, 49, 56]. Briefly, the compounds were dissolved in dimethyl sulfoxide (DMSO) (Sigma-Aldrich) and diluted in saline (0.9% NaCl) for 30 min under ultrasonic conditions.

Aqueous **2** and **2b** (0.01 mg/mL, pH 7.0) were dripped onto a formvar-coated copper grid. After thorough drying in air, the copper grid was kept in the dryer for 48 h. Then, the shape and size of the nanoparticles were observed with TEM and SEM. The details of this experiment are described in Additional file 1.

The particle size and surface zeta potential of the particles were measured using a particle size analyzer. The compounds were dissolved in ultrapure water with 1% DMSO. The concentration of the compounds in solution was 0.01 mg/mL, and the pH of the solution was pH 2.0, pH 5.5 or pH 7.4. The details of this experiment are described in Additional file 1.

### In vivo experiments

Male C57BL/6 mice were purchased from Beijing Vital River Laboratory Animal Technology Co., Ltd. The study was approved by the Institutional Animal Care and Use Committee of Capital Medical University, and the ethics number is AEEI-2018-174. The animals were cared for humanely throughout the animal studies. Male C57BL/6 mice were 8 weeks old at the beginning of the in vivo tumor metastasis assay. LLC cells were subcutaneously injected to form solid tumors. The subcutaneous tumors were implanted by injecting 0.2 mL of normal saline (NS) containing 1 × 10^6^ viable tumor cells under the skin into the right armpit of the mouse. When the tumor size reached approximately 5 mm in diameter (days 7–10 after implantation), the mice were randomly divided into the following treatment groups: **2b** (intraperitoneal dose: 1.0, 2.5 or 5.0 mg/kg/day, 9 consecutive days, 30 mice), NAMI-A (intraperitoneal dose: 35.0 mg/kg/day, 9 consecutive days, 10 mice), and vehicle: 20% 2-hydroxypropyl-*β*-cyclodextrin (intraperitoneal dose: 10 mL/kg/day, 9 consecutive days, 10 mice). The drugs were administered by intraperitoneal injection, and mice were weighed daily. 24 h after the last injection, the mice were weighed and killed with ether anesthesia, and the organs and tumor were immediately obtained.

### Degradation over time and toxicity studies of nanoparticles in vivo

Nanoparticle degradation assays were carried out with C57BL/6 mice implanted with LLC cell-derived tumors. Three mice in each group were sacrificed after 0.5 h, 1 h, 2 h, 8 h and 24 h. 2b was injected into the tail vein at a single dose of 5 mg/kg. Blood and organs were harvested immediately after the animals were sacrificed at the given time points. Blood was taken from the eye, added to tubes containing EDTA and centrifuged (2500 rpm for 5 min) to separate plasma and blood cells [57]. The heart, liver, spleen, kidneys, colon, lung, thymus and brain were collected and stored at − 80 °C until microwave digestion and analysis for the Ru content. The collected data were compared to those of a pharmacokinetic study of KP1019 {indazolium trans-[tetrachloridobis(1H-indazole)ruthenate(III)]}, which is a Ru(III) complex undergoing clinical trials [48].

In the toxicity study, 6 ICR mice implanted with S180 cell-derived tumors in each group were sacrificed on seven consecutive days. The drugs were administered via intraperitoneal injection at 1.0, 2.5 or 5.0 mg/kg/day, and the dose of cisplatin was 5 mg/kg. Blood was taken from the eye, added to tubes without anticoagulant and centrifuged (3000 rpm for 10 min at 4 °C) to obtain the serum. Serum Crea, Urea, ALT and AST levels were measured with a chemistry analyzer and the appropriate reagents (BS-600, Mindray, P.R. China). The heart, liver, spleen, kidneys, and brain were weighed.

## Supplementary Information

**Additional file 1.** Supplementary Information of a dual-targeting ruthenium nanodrug that inhibits primary tumor growth and lungmetastasis via the PARP/ATM pathway.

## Data Availability

All data generated or analyzed during this study are included in this published article [and its additional information files].

## Abbreviations

LLC: Lewis lung cancer
PDL1: Programmed cell death 1 ligand 1
CD31: Platelet endothelial cell adhesion molecule-1
hTF: Human transferrin
TF: Transferrin
TFR: Transferrin receptor
ICP-MS: Inductively coupled plasma mass spectrometry
^1^H NMR: ^1^H nuclear magnetic resonance
^13^C NMR: ^13^C nuclear magnetic resonance
MS: Mass spectroscopy
IR spectroscopy: Infrared spectroscopy
UV spectroscopy: Ultraviolet–visible spectroscopy
TEM: Transmission electron microscopy
SEM: Scanning electron microscopy
DMF: *N*,*N*-Dimethylformamide
bpy: 2,2′-Bipyridine
