# Supplementary material for: A dual-targeting ruthenium nanodrug that inhibits primary tumor growth and lung metastasis via the PARP/ATM pathway
Source: J Nanobiotechnology. 2021 Apr 23;19:115. doi: 10.1186/s12951-021-00799-3 (PMC8063440; doi:10.1186/s12951-021-00799-3)
Supplement: Supplementary file 1 — Additional file 1. Supplementary Information of a dual-targeting ruthenium nanodrug that inhibits primary tumor growth and lungmetastasis via the PARP/ATM pathway. [file 12951_2021_799_MOESM1_ESM.docx]

**Support Information of Synthesis, characterization, anti-tumor and anti-metastatic effects of novel ruthenium nanoparticles**

*Yu Lu B.S ^a b^, Di Zhu B.S ^a b^, Lin Gui PhD ^a b^, Yuanming Li B.S ^c^. Wenjing Wang PhD ^d^, Jiawang Liu PhD ^e^ and Yuji Wang PhD ^a b^ **

1. Synthesis

**General procedure for the synthesis of ruthenium complexes**

**Synthesis of 2a**

Forty-four milligrams (0.2 mmol) of ligand 2 and 104 mg Ru(bpy)_2_Cl_2_ were dissolved in 20 mL of EtOH/H_2_O. The reaction mixture was refluxed for 4 h. After 4 h, the mixture was concentrated at reduced pressure and purified with neutral alumina column chromatography, producing 88 mg of **2a** as a red powder (yield, 70.4%).

**Synthesis of 2b**

One hundred ten milligrams (0.5 mmol) of ligand 2 and 176 mg (NH_4_)_2_RuCl_6_ (0.5 mmol) were dissolved in 30 mL of N,N-dimethylformamide (DMF). The reaction mixture was refluxed for 24 h. After 24 h, the mixture was concentrated at reduced pressure and purified with neutral alumina column chromatography, producing 104 mg of 2b as black powder (yield, 45.8%).

1.1. Synthetic procedures

ⅴ

ⅳ

ⅲ

ⅱ

ⅰ

**Figure S1. Synthesis route of Ru complexes.**

Notesⅰ MeOH, H_2_SO_4_ and H_2_O; ⅱ KMnO_4_, MeOH; ⅲ 2M NaOH, MeOH ⅳ (NH_4_)_2_Ru(Ⅳ)Cl_6_, DMF, 80℃, 24h;, , H_2_O; ⅴ Ru(Ⅱ)(bpy)_2_Cl_2_, EtOH, H_2_O, room temperature, 24h.

**1.2. Synthesis of 2**

10 g (49 mmol) *L*-Trp, 20 mL of methanol, and 0.4 mL sulphuric acid was added. The reaction mixture was stirred at room temperature for 4 h. TLC is used to monitor the reaction. After 4 h, the reaction mixture is adjusted to pH 6 by adding concentrated aqueous ammonia and then kept statically for 0.5 h. The formed precipitates were collected by filtrated. The white solid is **(S)-2,3,4,9-tetrahydro-1H-pyrido[3,4-b]indole-3-carboxylic acid** weighed 10.27 g. Calculate the yield is 96.5%. ESI-MS: 217 [M+H]^+^. ^1^H NMR(300 MHz,*d*_6_-DMSO), δ(ppm) = 14.12 (s,1H), 11.20 (s,1H), 10.00 (1H,s) , 7.50 (d, 1H, *J* = 7.7), 7.37 (d, 2H, *J* = 7.98Hz), 7.11 (t, 1H, *J* = 7.31Hz), 7.02 (t, 1H, *J* = 7.23Hz), 4.48 (dd, 1H, *J* = 4.92Hz), 4.38 (s,1H) , 4.03 (dd, 1H, *J* = 7.12Hz), 3.31 (1H, dd, *J* = 6.87Hz), 3.04 (dd, 1H, *J* = 8.62Hz).

6 mL of SOCl_2_ is added dropwise to 80 ml of anhydrous methanol at 0℃. And the reaction mixture is stirred at 0℃ for 0.5 h and then (S)-2,3,4,9-tetrahydro-1H-pyrido[3,4-b]indole-3-carboxylic acid 4.32g {20 mmol} is added. The reaction mixture is stirred at room temperature for another 24 h and TLC is used to monitor the reaction. The reaction mixture is concentrated under vacuum. The resulting crude product is dissolved in 8 ml of methanol and then dried at reduced pressure for three times. The residue is again suspended in ether and concentrated under vacuum for three times. The pure product was obtained by column chromatography. The colorless powder is **methyl (S)-2,3,4,9-tetrahydro-1H-pyrido[3,4-b]indole-3-carboxylate** weighed 1.65 g. Calculate the yield is 35.9%. ESI-MS (m/e): 231 [M + H]^+^. ^1^H NMR（300 MHz, *d_6_*-DMSO）, δ = 11.20 (s, 1H), 10.15 (s, 1H), 7.48 (d, 1H, *J* = 7.8 Hz), 7.38 (d, 1H, *J* = 8.1 Hz), 7.12 (t, 1H, *J* = 7.5 Hz), 7.02 (t, 1H, *J* = 7.5 Hz), 4.62 (dd, 1H, *J*_1_ = 5.1 Hz, *J*_2_ = 9.9 Hz), 4.39 (s, 2H), 3.83 (s, 3H), 3.30 (dd, 1H, *J*_1_ = 14.7 Hz, *J*_2_ = 4.5 Hz), 3.07 (dd, 1H, *J*_1_ = 15.9 Hz, *J*_2_ = 10.2 Hz).

methyl (S)-2,3,4,9-tetrahydro-1H-pyrido[3,4-b]indole-3-carboxylate 460 mg (0.2 mmol) was dissolved in 15 mL of acetone to get solution A. And solution B was made by dissolving KMnO_4_ 474 mg (0.4 mmol) in 10 mL of water and then transferred B to A drop by drop at 0 ℃. The reaction mixture was stirred at room temperature overnight and TLC was used to monitor the reaction. Added ethanol to terminate the reaction. The resulting crude product was filtered to remove MnO_2_ and the solvent was removed in vaccum from the filtrate. The pure product was obtained by column chromatography. The Pale yellow powder is **methyl 9H-pyrido[3,4-b]indole-3-carboxylate** weighed 174 mg. Calculated yield is 38.5%. ESI-MS (m/e): 227 [M + H]^+^. ^1^H NMR (300 MHz, *d_6_*-DMSO), δ = 12.09 (s, 1H), 8.97 (s, 1H), 8.94 (s, 1H), 8.41 (d, 1H, *J* = 7.8 Hz), 7.68 (d, 1H, *J* = 8.1 Hz), 7.61 (t, 1H, *J* = 7.5 Hz), 7.32 (t, 1H, *J* = 7.2 Hz), 3.91 (s, 3H).

Dissolve methyl 9H-pyrido[3,4-b]indole-3-carboxylate 100mg (0.044mmol) in 10mL of methanol at 0℃. Adjusting the mixture to pH 12 by adding 2N NaOH. The reaction mixture is stirred at 0℃ for 6h When TLC plate indicates a complete disappearance of the raw material, quench the reaction by adjusting the reaction to pH 7 with aqueous KHSO_4_ solution. After filtration, the filtrate was concentrated under reduced pressure. After a small amount of water is added to the syrup, a brick red solid is washed out. The title compound was obtained after filter and dry. The brick red powders weighed 36.6 mg. The filtrate was adjusted to pH 2 by saturated aqueous KHSO4 solution, and extracted with ethyl acetate(20 mL*3). The organic phase was washed with saturated aqueous NaCl solution (15mL*3). The organic layer was dried over anhydrous Na_2_SO_4_ for 2h. The title compound was obtained after filtration and evaporation under reduced pressure. The colorless powder is **9H-pyrido[3,4-b]indole-3-carboxylic acid(2)** weighed 16.6 mg. Calculateed gross yield is 56.7%. ESI-MS (m/e): 212.98 [M + H]^+^. ^1^H NMR（300 MHz, d_6_-DMSO）, δ = 12.20 (s, 1H), 9.00 (s, 2H), 8.45 (d, 1H, *J* = 7.8 Hz), 7.71 (d, 1H, *J* = 8.1 Hz), 7.64 (t, 1H, *J* = 7.5 Hz), 7.35 (t, 1H, *J* = 7.2 Hz). ^13^C NMR (800 MHz,*d*_6_-DMSO), δ(ppm) = 166.54, 141.95, 137.60, 136.38, 132.91, 129.69, 129.01, 122.95, 121.24, 120.97, 118.05, 113.00. IR 3500-3200 cm^-1^, 3067 cm^-1^, 2922 cm^-1^, 1732 cm^-1^, 1661 cm^-1^, 1636 cm^-1^, 1598 cm^-1^, 1497 cm^-1^, 1343 cm^-1^, 1261 cm^-1^.

**1.3. Synthesis of 2a**

44 mg (0.2 mmol) of ligand 2, 104 mg Ru(bpy)_2_Cl_2_ was dissolved in EtOH / H2O. The reaction mixture was heated at 60℃ for 4h. After 4h, the mixture was concentration at reduced pressure and was purified by neutral alumina column chromatography, to give a red solid powder (88 mg, 70.4%). ESI/MS（m/z）: 625.69 [M-Cl]^+^；^1^H NMR (300 MHz,*d*_6_-DMSO), δ（ppm）= 9.65 (1H, dd, 12.00Hz), 9.57 (s, 1H), 9.15 (d, 1H, *J* = 39.72Hz), 8.59 (t, 1H, *J* = 7.23Hz), 8.51 (d, 1H, *J* = 7.62Hz), 8.42 (d, 2H, *J* = 7.53Hz), 8.34 (d, 2H, *J* = 7.65Hz), 8.03 (d, 3H, *J* = 7.47Hz), 7.81 (t,,1H, *J* = 5.31Hz), 7.74 (t, 1H, *J* = 5.31Hz), 7.63 (d, 2H, *J* = 3.30Hz), 7.48 (d,2H, *J* = 3.30Hz), 7.28 (m, 2H), 7.14 (d, 1H, *J* = 5.67Hz), 7.09 (d, 2H, *J* = 3.30 Hz). ^13^C NMR(300 MHz,*d*_6_-DMSO), δ(ppm) = 157.92, 157.48, 157.32, 156.18, 155.45, 153.25, 152.98, 149.37, 139.33, 139.21, 138.48, 138.15, 128.32, 127.62, 127.36, 127.17, 124.86, 124.51, 124.03, 123.58, 48.93. IR 3426 cm^-1^, 3067 cm^-1^, 3045 cm^-1^, 2958 cm^-1^, 2932 cm^-1^, 2820 cm^-1^, 1957 cm^-1^, 1642 cm^-1^, 1618 cm^-1^, 1599 cm^-1^, 1458 cm^-1^, 1416 cm^-1^.

**1.4. Synthesis of 2b**

110 mg (0.5 mmol) of ligand 2, 176 mg (NH_4_)_2_RuCl_6_ (0.5 mmol) was dissolved in DMF (N,N-dimethylformamide). The reaction mixture was heated at 80℃ for 6h. After 6h, the mixture was concentration at reduced pressure and was purified by neutral alumina column chromatography, to give a black solid powder (104 mg, 45.8%). ESI-MS (m/e): 455.8 [M + H]^+^. ^1^H NMR（300 MHz, *d_6_*-DMSO）, δ = 12.20 (s, 1H), 9.00 (s, 2H), 8.45 (d, 1H, *J* = 7.8 Hz), 7.71 (d, 1H, *J* = 8.1 Hz), 7.64 (t, 1H, *J* = 7.5 Hz), 7.35 (t, 1H, *J* = 7.2 Hz). ^13^C NMR(800 MHz,*d*_6_-DMSO), δ(ppm) = 163.19, 143.97, 136.41, 132.38, 132.06, 130.05, 124.17, 122.42, 120.74, 119.48, 113.89, 49.08. IR 3219 cm^-1^, 3059 cm^-1^, 2924 cm^-1^, 2845 cm^-1^, 1621 cm^-1^, 1599 cm^-1^, 1342 cm^-1^, 1294 cm^-1^.

**Figure S2 ^1^HNMR of 2**

**Figure S3 ^13^CNMR of 2**


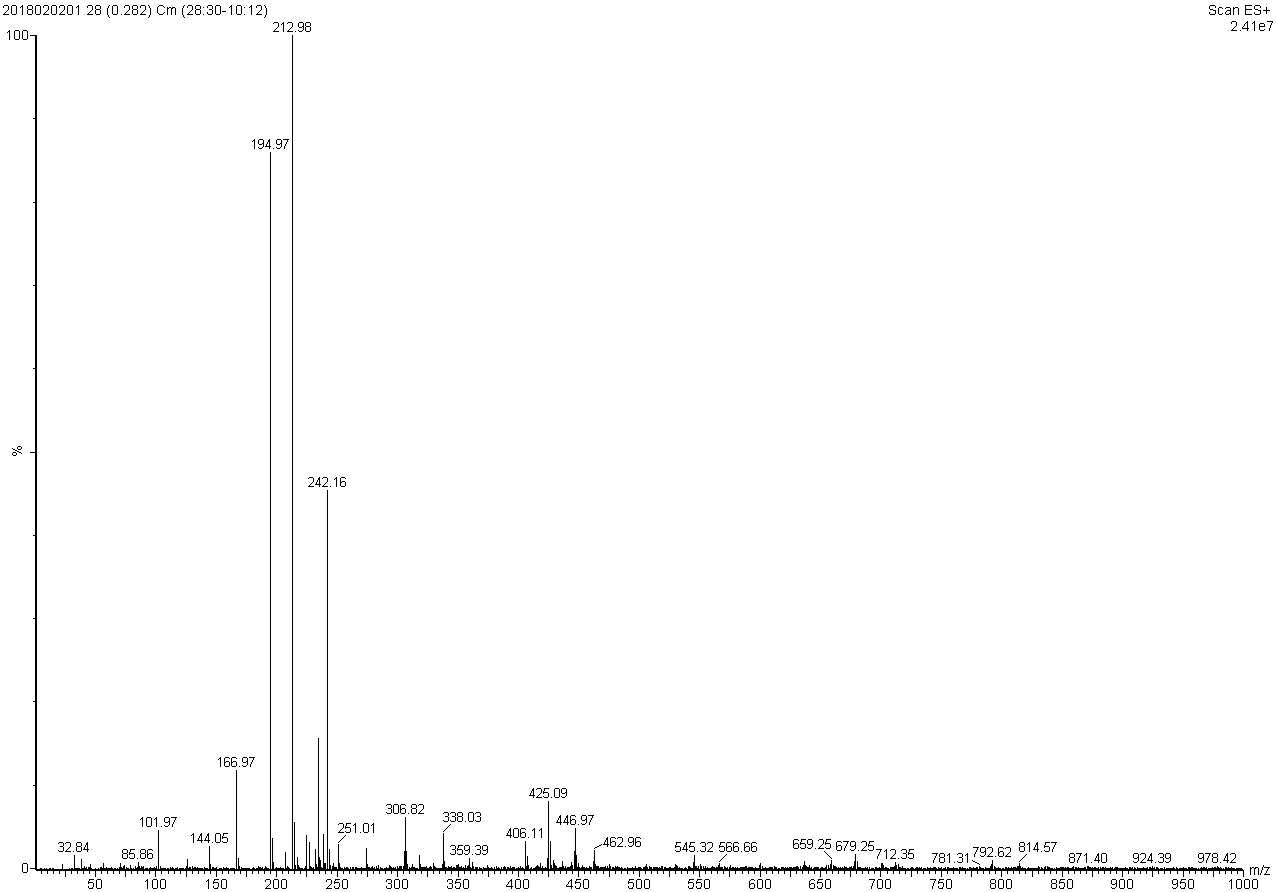


212.98

**Figure S4 MS of 2**


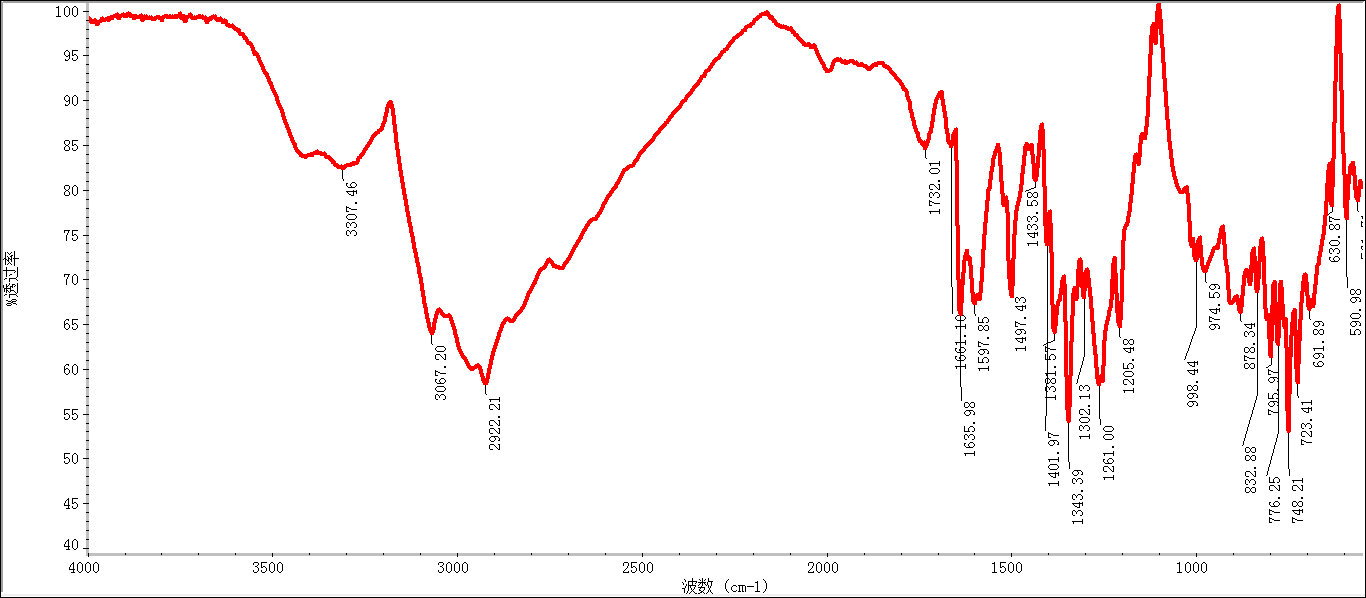


**Figure S5 IR of 2**

**Figure S6 ^1^HNMR of 2a**

**Figure S7 ^13^CNMR of 2a**

625.69

**Figure S8 MS of 2a**


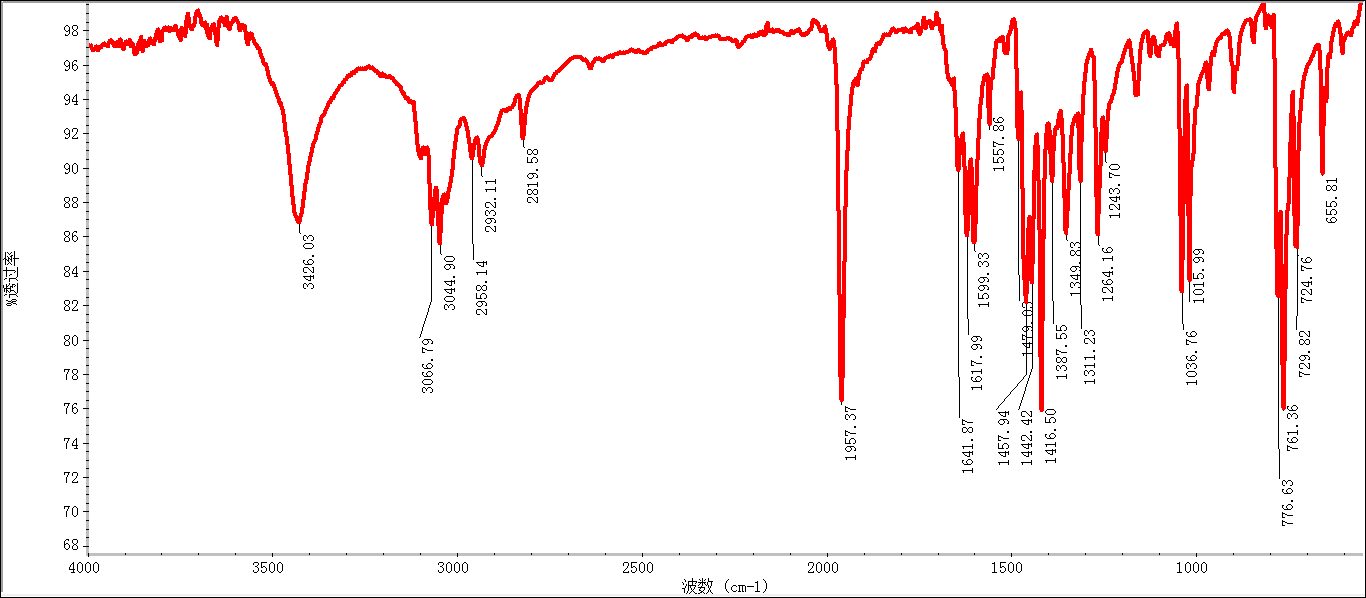


**Figure S9 IR of 2a**

**Figure S10 ^1^HNMR of 2b**

**Figure S11 ^13^CNMR of 2b**

455.8

**Figure S12 MS of 2b**


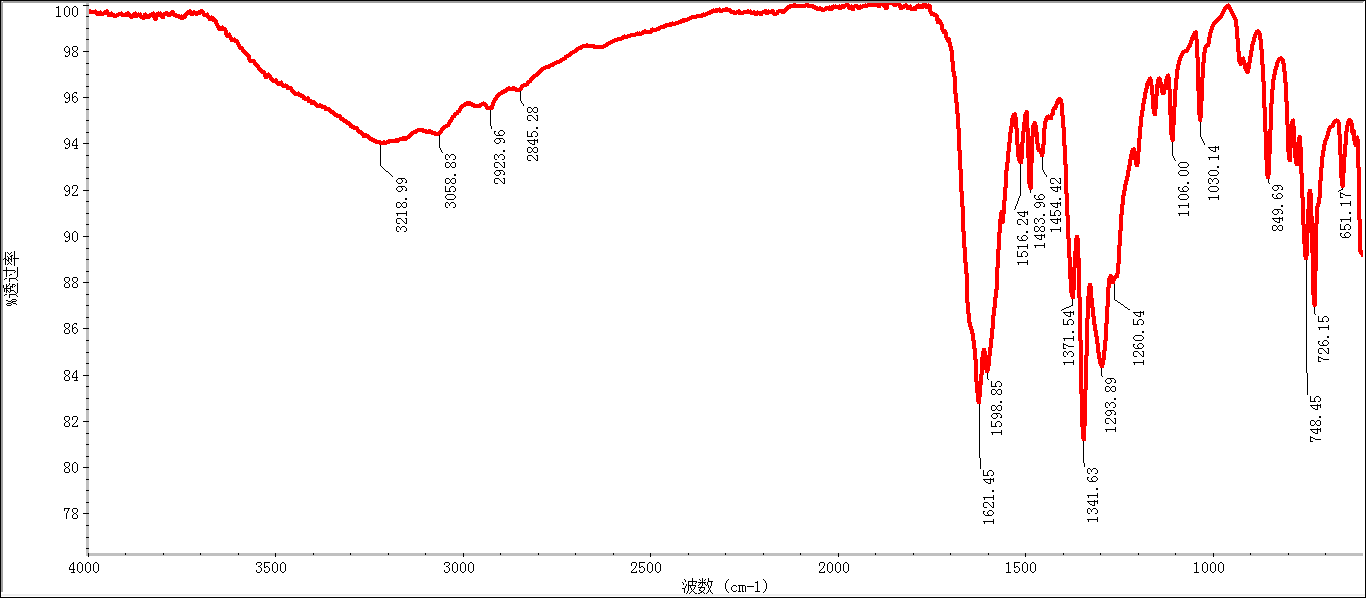


**Figure S13 IR of 2b**

**2. Materials and reagents**

methanol, sulphuric acid, ammonia, SOCl_2_, Acetone, KMnO_4_, Ethanol, NaOH, KHSO_4_, ethyl acetate, NaCl, Na_2_SO_4_, dimethylbenzene, DMF (N,N-dimethylformamide) are purchased from Beijing Chemical Reagent Company. *L*-Trp (BeiJingBoMaiJie Technology Co,Ltd), (NH_4_)_2_RuCl_6_ (Macklin,  A801742-1g), , RPMI 1640 medium (Gibco, 31800-022), FBS (Corning, 35-076-CV), Penicillin G Sodium salt (100 U/mL) (Solarbio, P8420), Streptomycin Sulfate (Solarbio, S8290), DMEM medium (Gibco, 12100-046), MTT ((3-(4,5-dimethylthiazol-2-yl)2,5-dipHenyl-tetrazolium bromide) (Aladdin, CAS:298-93-1), 0.25% trypsin (Hyclone, KGY0012), Cisplatin (Sigma-Aldrich, CAS:15663-27-1), DMSO (Hyclone, 57-68-5), PBS (KeyGEN BioTECH, KGB5001), Annexin V-FITC/PI kit (Beyotime), Annexin V-PE/Hoechest kit (Beyotime), Cell lysis buffer for Western and IP (Beyotime), 4% paraformaldehyde solution, formvar-coated copper grid, ct-DNA (calf thymus from Sigma-Aldrich), Human transferrin (Sigma-Aldrich, T2252-100MG), T75 cell culture flask (Corning), nuclear mitochondrial separation kit (*Beyotime*), BCA protein assay reagent (*Beyotime*), Neutral gum (Macklin, N861409-100ml) , BSA (Amresco, A7030), Tunel detection kit (fluorescence) (*GenePool*, GPB1829), Anti-fluorescence Attenuation Seal Tablets (with DAPI) *(Solarbio*, S2110), EDTA (pH 9.0) antigen repair solution (*Genepool*, GPB1837), CD31 (*Abcam*, Ab28364), Goat Anti-Rabbit IgG H&G (Biotin) (Abcam, ab6720), Streptavidin(HRP) (Abcam, ab7403), DAB Kit(20X) (Genepool, GPP1823), NAMI-A (MCE, GY02983), HP-β-CD (Macklin, H811091-100 g).

A549, 95D, S180, A549-TAX, L02, LLC cells were purchased from KeyGEN BioTECH.

Male C57BL/6 mice were purchased from Beijing Vital River Laboratory Animal Technology Co., Ltd. The study was approved by Institutional Animal Care and Use Committee (IACUC), and the Ethics number is AEEI-2018-174.

3. Nano self-assembly properties

To explore the nano self-assembly properties of 2b and its ligand in solution and in solid state, Particle Size Analyzer, transmission electron microscopy (TEM) and scanning electron microscopy (SEM) were applied.

The aqueous 2 and 2b (0.01 mg/mL, pH 7.0) were dripped onto a formvar-coated copper grid. After thorough drying in air, the copper grid was kept in the dryer for 48h. Then the shape and size of nanoparticles were obeserved with a TEM (JSM-6360 LV; JEOL, Tokyo, Japan). Each determination was performed with triplicate copper grids. TEM images were recorded at an electron beam accelerating voltage of 80 kV and on a 6,000–400,000× digitally enlarged imaging plate (Gatan Bioscan Camera Model 1792; Gatan, Inc., Pleasanton, CA, USA) with 20 eV energy windows.

To examine the particle size of 2 and 2b, we also used SEM (50 kV; JEM-1230; JEOL). Lyophilized powders formed from aqueous compounds (0.01 mg/mL, pH 7.0) were attached onto a copper plate with double-sided tape (Euromedex, Souffelweyersheim, France). On a JEOL JFC-1600 Auto Fine Coater, the specimens were coated with 20 nm gold–palladium. At 15 kV, 30 mA, and 200 mTorr (argon), the coater was operated for 60 s. The feature and size distribution of the particles were visualized by examining. Each measurement was performed with triplicate copper plates. The images were recorded on a 100–10,000× digitally enlarged imaging plate of Gatan Bioscan

Camera Model 1792 with 20 eV energy windows

The particle size and surface zeta potential of the particles in ultrapure water of pH 2.0, pH 5.5 and pH 7.4 were measured using a Particle Size Analyzer (ZetaPlus S/N 21394; Brookhaven Instruments Corporation, Holtsville, NY, USA) by using a dynamic light scattering (DLS) model. The size measurement was repeated for three runs per sample. The surface zeta potential of the particles was measured using a ZetaPlus Potential Analyzer (ZetaPlus S/N 21394) with a BIC Zeta Potential Analyzer. The concentration of the solution in ultrapure water of pH 2.0 and in PBS of pH 7.4 was 0.01 mg/mL, and the testing temperature was 25℃. The potential change for 48 hours is continuously recorded. The zeta potential mea­surement was repeated for three runs per sample, and the data were calculated automatically using the software from the electrophoretic mobility based on Smoluchowski’s formula.


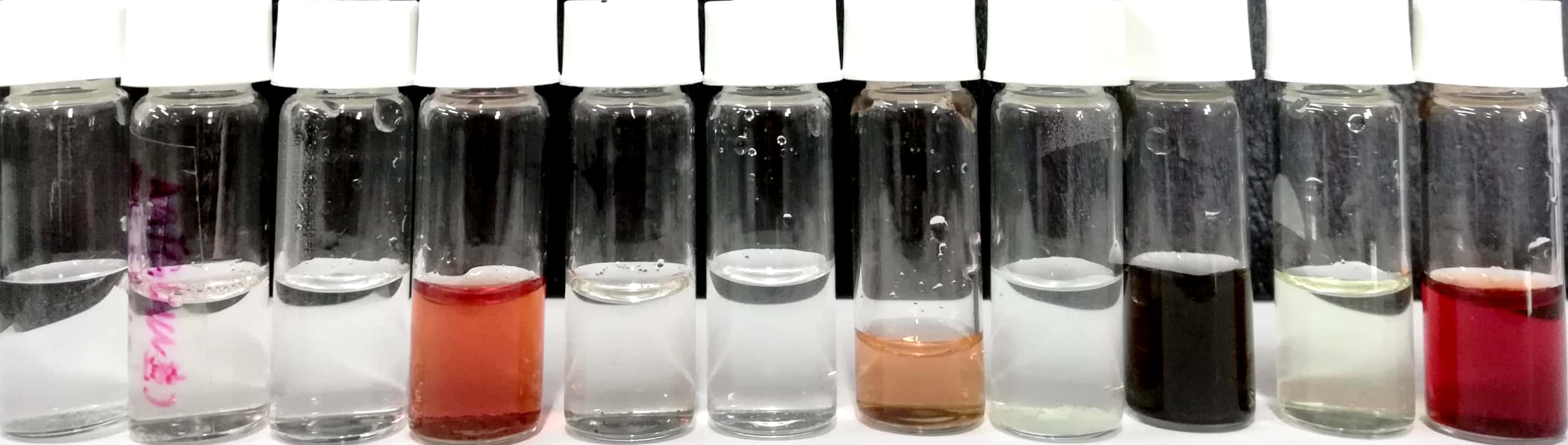


Figure S14. 2 (left) and 2b (right) was dissolved at 1 mg/ml in water.

**4. DNA interactions studies**

The concentration of ct-DNA (calf thymus from Sigma-Aldrich) was determined before the experiment began. The DNA concentration dissolved in PBS (pH 7.4) was determined by ultraviolet spectrophotometry analysis using molar absorption coefficient of 6600.0 mol^-1^·L·cm^-1^ at 260.0 nm.

To determine the binding constants (K_b_) of **2b** with DNA, **2b** (12.5 μM) was titrated with ct-DNA in PBS (pH 7.4). **2b** was diluted to 25 μ M with 1% DMF/PBS. The volume of colorimetric dish was 4 mL and the thickness was 1 cm. Using PBS or corresponding concentration of DNA as blank control. 3mL compound solution was added to the colorimetric dish, and DNA solution was dripped into it. The absorbance values are recorded in the wavelength range of 220~600nm. The binding constant K_b_ formula is calculated by Benes-Hildebrand equation.

[DNA]/(ε_a_-ε_f_) = [DNA]/(ε_b_-ε_f_) + 1/K_b_(ε_b_-ε_f_)

ε_a_ is the apparent molar extinction coefficient (ε_a_ = Aobserved/[complex]). ε_b_ is the extinction coefficient of the DNA bound complex, and ε_f_ is the extinction coefficient of the free extinction. According to the equation, the binding constant of the complex is calculated by slope and intercept.

**5. Human transferrin binding**

A stock solution of human transferrin was prepared in PBS (pH 7.4) to be used for different set of experiments. The hTF concentration was determined by absorption spectrophotometric analysis using a molar absorption coefficient of 113000 mol·L·cm^-1^at 289.0 nm.

Shimadzu F-2500 spectrometer (Tokyo, Japan) were used to recorded the emission spectra. The volume of colorimetric dish was 4 mL and the thickness was 1 cm. Complexes were dissolved in 1% DMSO/H_2_O. When the fluorescence emission spectrum was measured (excitation wavelength 280 nm), the concentration of hTF was kept and the complexes was gradually added into until the fluorescence intensity no longer changed. The fluorescence intensity of transferrin was quenching at 310 nm. Please refer to the supplementary information for details of data analysis.

F_0_/F = 1+Ksv*[Q] (1)

Kq = Ksv/τ_0_ (2)

log [(F0-F)/F] = log K + n log [Q] (3)

Where F_0_ denotes fluorescence intensity of native hTF at 310 nm, F denotes fluorescence intensity of hTF during titration, Ksv is the Stern-Volmer constant, and [Q] is the concentration of complexes.

The experiments were analyzed by the classical Stern-Volmer equation (1) and modified Stern-Volmer (3). According to equation (1) and (2), the apparent bimolecular quenching rate constant K_q_ can be calculated. τo is the average integral fluorescence life time of tryptophan which is 5.78*10^-9^. When K_q_ is greater than 2*10^10^, the quenching mechanism is by static, otherwise it is by dynamic. In the equation (3), we can find out the number of binding sites (n) and the binding constant (K_b_).

**6. FT-IR for human transferrin (hTF) and DNA to complex 2b**

The hTF concentration was determined by absorption spectrophotometric analysis using a molar absorption coefficient of 113000 mol·L·cm^-1^at 289.0 nm. And the concentration of ct-DNA was determined using molar absorption coefficient of 6600.0 mol^-1^·L·cm^-1^ at 260.0 nm. Afterwards, different volumes of complex 2b solution was added to 100 μL of hTF or DNA solution. hTF or DNA were incubated with complexes at a 1:1, 2:1 and 4:1 ratio in PBS (pH 7.4 ) at 37°C for 15 min on a shaker. After incubation, 10 μL of the mixed solution was dropped onto the detector. FTIR spectra was recorded using ATR unit *on* a Thermo scientific Nicolet iS5 spectrometer.


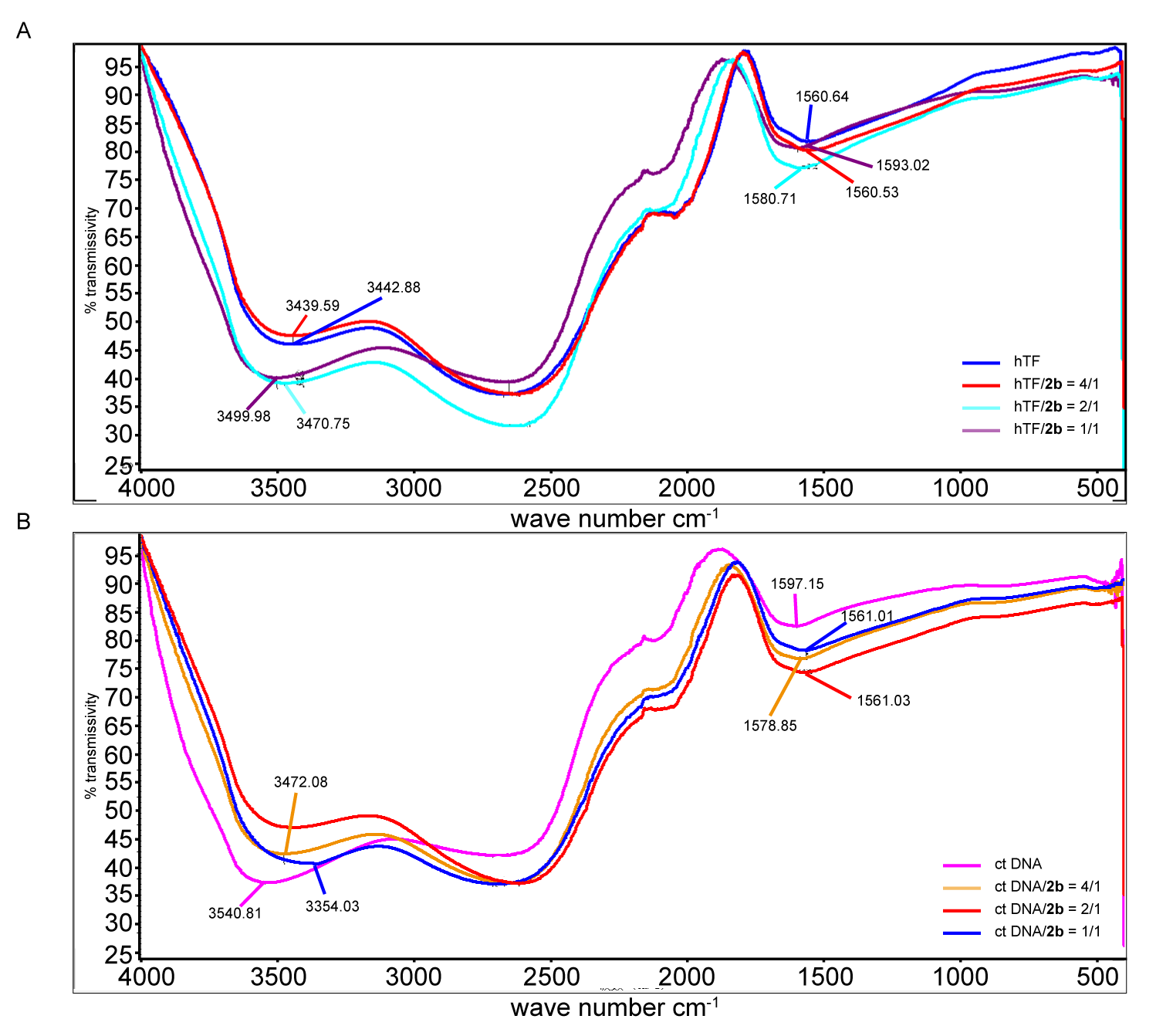


PeakⅡ

PeakⅠ

Figure S15. FT-IR spectra for human transferrin (hTF) and DNA to complex 2b. (A) FT-IR spectra for hTF to complex 2b in different proportions. (B) FT-IR spectra for ct-DNA to complex 2b in different proportions.

7. *In vitro* cytotoxic study

A549 (human lung carcinoma cell), 95D (Human lung cancer with high metastasis), S180 (mice sarcoma S180 cell ), A549-TAX (Paclitaxel resistant cells in human lung cancer) were maintained in RPMI 1640 medium supplemented with 10% FBS, penicillin (100 U/mL), and streptomycin (100 μg/mL). LLC (Lewis lung cancer cell), L02 (normal human embryonic hepatocytes) were maintained in DMEM medium supplemented with 10% FBS, penicillin (100 U/mL), and streptomycin (100 μg/mL). The cells were maintained in a humidified atmosphere of 5% CO2 at 37°C. The medium was renewed every 2 days. The proliferation of cancer cell lines and one normal cell (L02 cell line) was determined with MTT [3-(4,5-dimethylthiazol-2-yl)-2,5-diphenyltetrazolium bromide] assay. In brief, cancer cell lines or L02 cell line in the logarithmic growth phase were digested with 0.25% trypsin, added to RPMI 1640 medium or DMEM medium supplemented with 10% FBS, plated into 96-well plates (3×10^3^cells/well), and incubated for 8 hours. The cells were treated with Ru compounds (final concentration: 3.125, 6.25, 12.5, 25, 50, and 100 μM), Cisplatin and (NH_4_)_2_Ru(Ⅳ)Cl_6_ were used as standard positive control drugs. Cells in the control wells also acquired the same volume of medium containing 0.1% DMSO. After 48 h, 25 μL solution of MTT in the PBS (5 mg/mL) was added, and the plates were incubated at 37°C for 4 hours. The supernatant was discarded and to each well 150 μL of DMSO was added. The optical density was read with Spectra Max M3 microplate reader (BioTek, Winooski, VT, USA) at 490 nm and 570 nm. The optical density (OD) value of compounds-treated well was compared with that of DMSO-treated well. The proliferation of cancer cell line or L02 cell line was represented with cell viability and IC_50_. Each measurement was performed in triplicate. Data are presented as mean ± SD.

Table S1 MTT cytotoxicity of Ru complexes at 48 h of drug exposure

| compounds | IC_50_（μM）± SD | | | | | | |
| --- | --- | --- | --- | --- | --- | --- | --- |
|  | S180 | | LLC | A549 | A549-TAX | 95D | L02 |
| (NH4)_2_Ru(Ⅳ)Cl_6_ | ＞100 | ＞100 | | ＞100 | ＞100 | ＞100 | ＞100 |
| NAMI-A | ＞200 | ＞200 | | ＞200 | ＞200 | ＞200 | ＞200 |
| 2 | 63.9±10.2 | ＞100 | | 79.39±5.6 | 84.32±5.8 | 61.60±6.9 | 77.23±8.4 |
| 2a | ＞100 | ＞100 | | ＞100 | ＞100 | ＞100 | ＞100 |
| 2b | 91.59±7.8 | 56.12±6.8 | | 13.48±4.5 | 59.69±6.6 | 57.03±6.0 | ＞100 |
| cisplatin | 1.6±0.2 | n.d. | | 9.7±1.5 | n.d | n.d | 43.56 |

7. Distribution of Ru *in vitro* and *in vivo*

To measure the cellular uptake of ruthenium, exponentially growing A549 cells were harvested and plated in T75 cell culture flask (Corning) at 100000 cells per flask. After incubation for 12h at 37 ℃，5% CO_2_, discarded the old medium and replaced it with a medium containing 2b (50 μM), vehicle. After treatment for 24 h, the medium was removed and the cells were washed three times with PBS. The cells were then harvested using a cell scraper. The cell suspension was centrifuged at 800g for 3min. The collected cells were isolated from the nucleus and mitochondria according to the nuclear mitochondrial separation kit (Beyotime) cell fractions were digested in H_2_O_2_ and HNO_3_(1/1) using MARS5 Microwave digestion system. Then fixed volume of the digested liquid to 10 mL with water to determine the content of ruthenium using Agilent Technologies 8800 ICP-MS Triple Quad. Ruthenium standard solution was purchased from Macklin, and rhodium^103^ was the internal standard. In the meantime, the cell fractions were added cell lysate with protease inhibitor to decompose the nucleus and mitochondria. Then sonicated for protein determination by the BCA (bicinchoninic acid) method using a BCA protein assay reagent (Beyotime). Data were expressed as Ru ng per mg protein. Results were reported as the mean ± SD (n = 3)

In the antitumor assay, the LLC tumor-bearing mice treated with 2b (5.0 mg/kg) were sacrificed by diethyl ether anaesthetized and dissected to immediately obtain and weight the tumor, liver, kidney, brain, spleen and heart. The biological samples less than 300 mg were digested in H_2_O_2_ and HNO_3_(1/1) using MARS5 Microwave digestion system. Then fixed volume of the digested liquid to 10 mL with water to determine the content of ruthenium using Agilent Technologies 8800 ICP-MS Triple Quad. Ruthenium standard solution was purchased from Macklin, and rhodium^103^ was the internal standard.


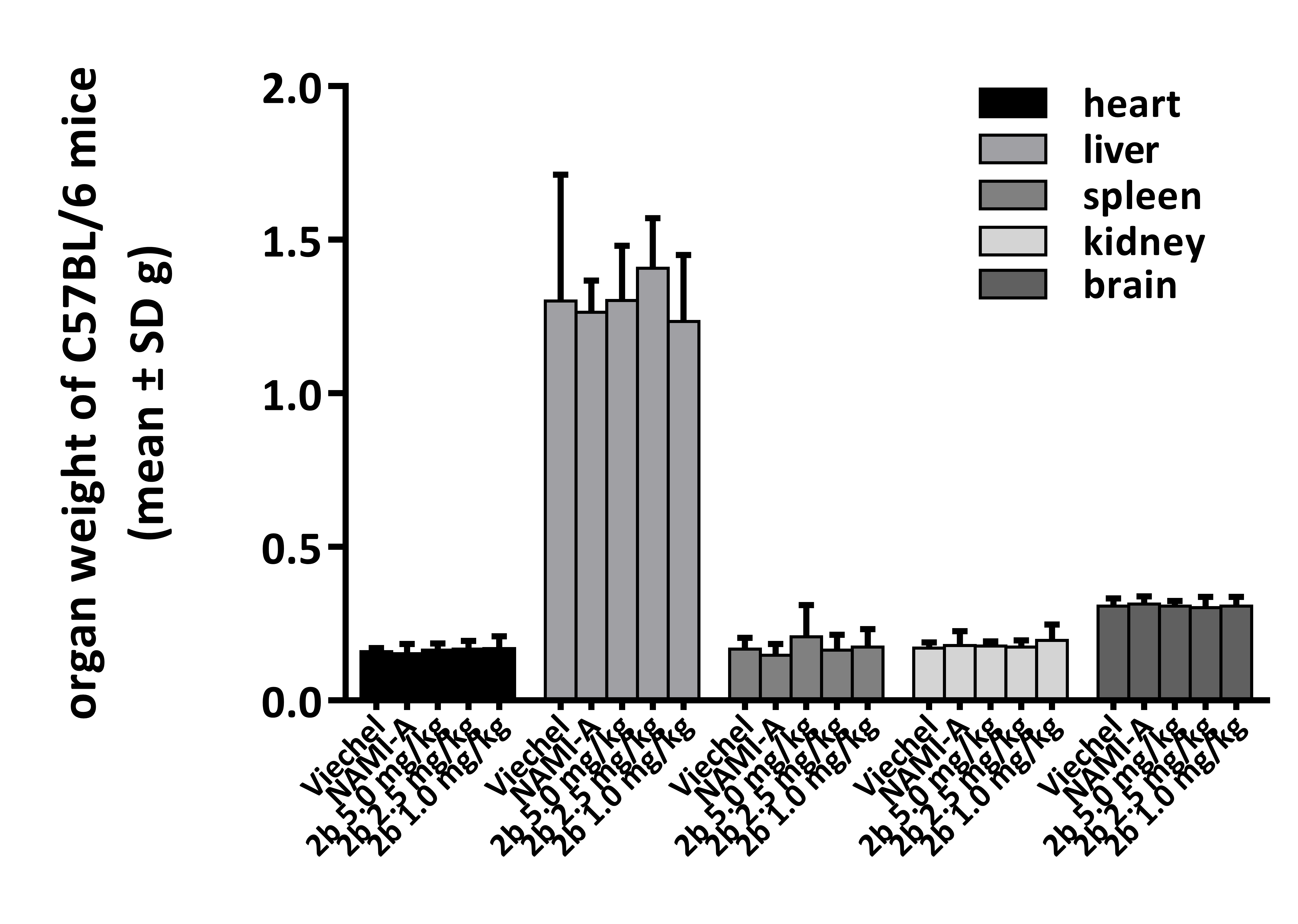


Figure S16. The organ weight of C57BL/6 mice.

8. Cell cycle and apoptosis

A549 cells were treated with 2b (final concentration: 10, 20, 50μM) for 48 h. Then the cells were harvested and centrifuged (5 min at 1000 g) and fixed in 2 mL of 70% aqueous ethanol (v/v). After an incubation of 12 h at 4℃, cells were centrifuged (10 min at 800 g) and washed twice with ice-cold PBS. Preparation of PI staining solution according to the kit specification. Cells were resuspended with 500μL staining solution containing PI and RNase A. Then the cells were incubated in darkness for 30 minutes and analyzed by EPICS@XL flow cytometry (BECKMAN COULTER, Germany). The number of cells analyzed for each sample was more than 10,000, and the experiments were repeated three times under identical conditions.

A549 cell apoptosis was detected by Annexin V-FITC/PI kit (Beyotime) and Annexin V-PE/Hoechest kit (Beyotime). For confocal microscopy, A549 cells were cultured in 35 mm glass-bottom dishes (Corning) and treated with 2b at different concentration for 48 h. After washing with PBS,1 ml Hoechest staining solution was added to each dish. After incubation for 20 min at 37 ℃, discard the dye and wash it with PBS. Then the cells were stained with Annexin V-PE for 20 min at room temperature and observed immediately with a laser confocal microscope (TCS SP8 STED, Leica, Germany). For flow cytometry analysis, A549 cells were trypsinized and washed twice with PBS. Then the cells were stained with Annexin V-FITC for 20 min and PI for 5 min. The fluorescence intensity was measured by a LSRFortessa SORP flow cytometry (Becton, Dickinson and Company). The FL-1 channel is FITC-A, and the FL-2 channel is PI-A. Cells in Q2 and Q4 area were considered apoptotic.

9. Western Blot analysis

After treatment for 48 h, the cells were harvested using a cell scraper. The cell suspension was centrifuged at 800g for 3 min. Then the protein was collected according to instructions of Cell lysis buffer for Western and IP (Beyotime). After quantification of the protein, added 60ug protein sample to each pore of SDS-PAGE gel electrophoresis. Then transferred to PVDF membrane. The membrane was blocked with 5% milk blocking buffer for 1 h. Then the membrane was incubated with antibodies at 4 ℃ for overnight. And the secondary antibody labeled by HRP was incubated for 1 h. the PVDF membrane was immersed in the ECL chromogenic solution () for 1 min and exposed in the dark chamber. The gray value of band was read by Quantity One v.4.62 software.

10. Immunofluorescence and immunohistochemistry

After the tumor tissue was removed from the mice, it was immediately fixed by 4% paraformaldehyde solution and the paraffin section was prepared. Then paraffin sections of tumor tissue were dewaxed to water and and the antigen was repaired. The paraffin sections were blocked with 5% milk blocking buffer for 1 h. Then it was incubated with antibodies at 4 ℃ for overnight. And the secondary antibody labeled by HRP or fluorescent material was incubated for 1 h, the paraffin sections were exposed to haematoxylin or DAPI in the dark. Then, the paraffin sections were observed by Slice Scanner. The terminal deoxynucleotidyl transferase-mediated dUTP-biotin nick end labeling (TUNEL) assay was used to detect the apoptosis of tumor issue according to the protocol. The degree of apoptosis was calculated by the IOD of TUNEL-positive cells. The expression of CD31 was calculated by the IOD of brown section.


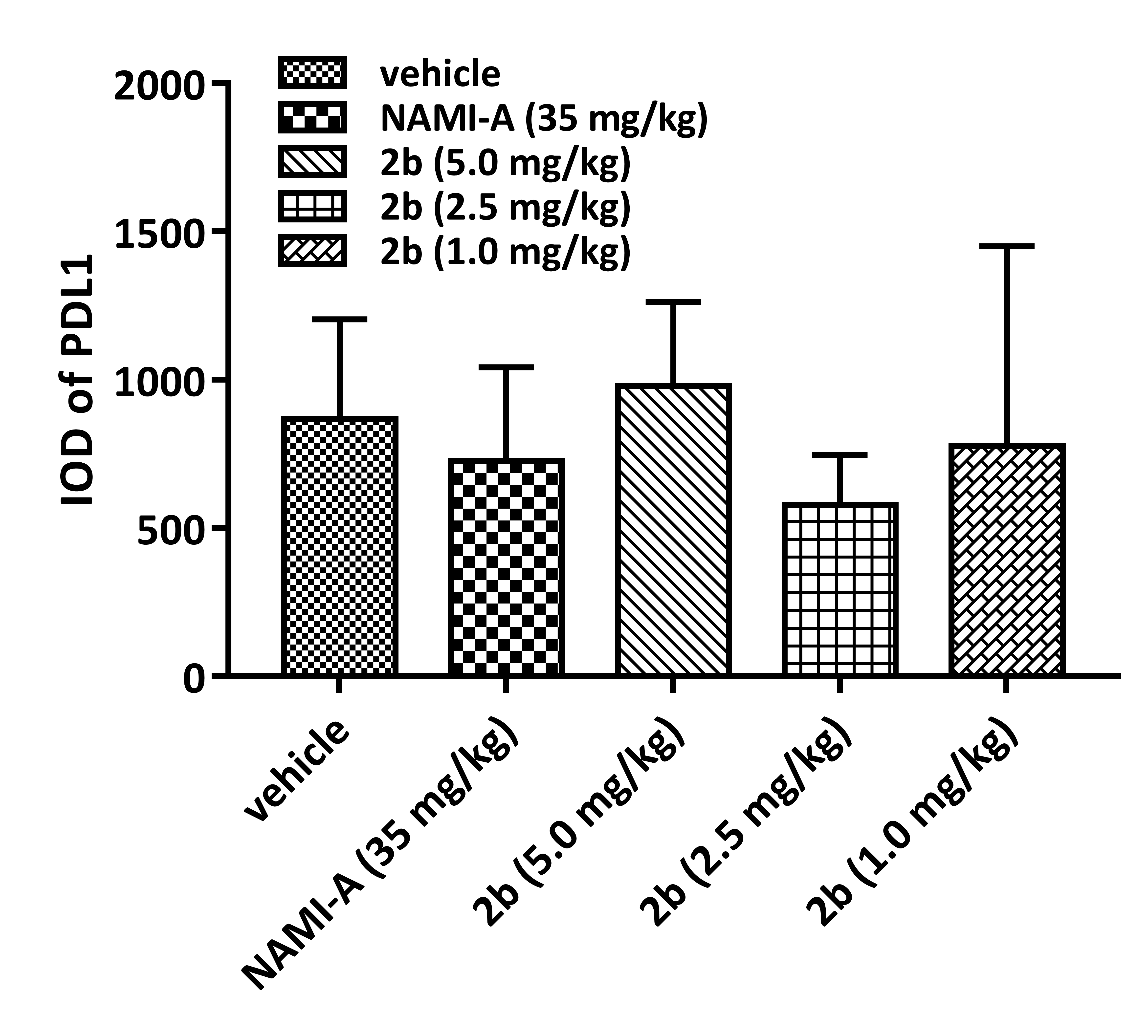


Figure S17. The expression of PDL1 in C57BL/6 mice.

Figure S18. The content of Ru in heart and brain


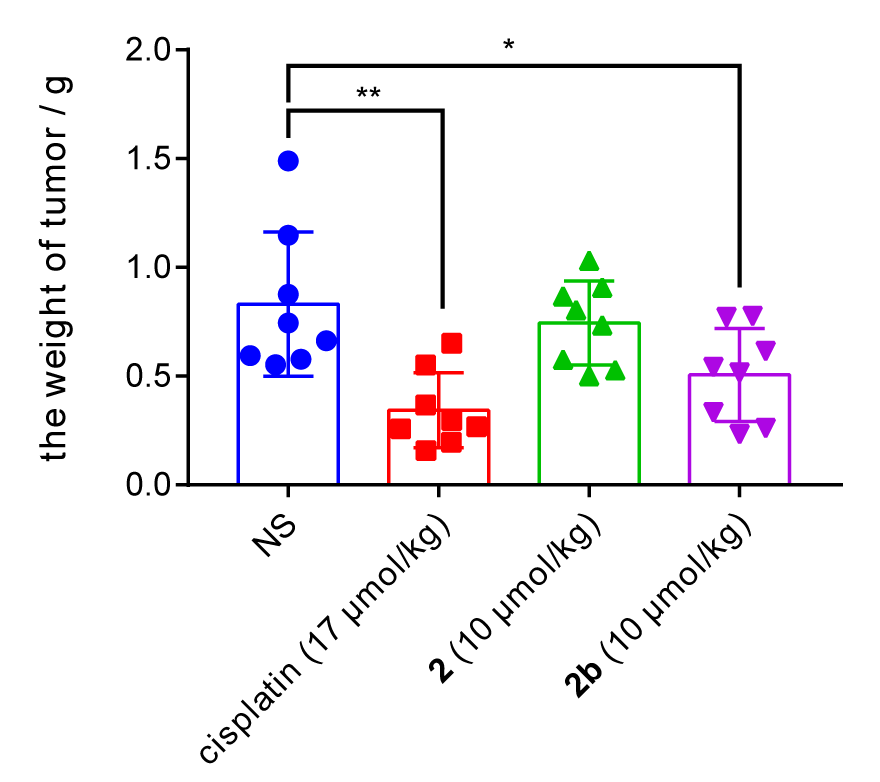


Figure 19. the tumor weight of ICR mice implanted with S180 tumor.

Figure S20. (a) The organ weight of S180 mice. (b-e) the serum levels of ALT, AST, Crea and Urea.

Capillary tube formation assay

Matrigel was thawed on ice at 4°C, and then 250 μL was added to the wells of 24-well plates (Corning) and polymerized for 45 min at 37°C. HUVECs were harvested and resuspended in 10% FBS-containing DMEM medium. The concentration of cell suspensions was adjusted to 2*10^5 cells / mL. 500 μL cell suspensions were plated onto Matrigel-coated wells. 2b and NAMI-A were added to the plates. Each well was then photographed once every 2 h. The formation of tubes was measured for further quantification of angiogenic activity of endothelial cells. The total tube length in three random view-fields per well was measured by Image J software.
